# Inspiratory Muscle Training on Exercise Capacity, Dyspnoea and Health Status in Pulmonary Hypertension: A Randomised Controlled Trial

**DOI:** 10.1111/resp.70054

**Published:** 2025-05-26

**Authors:** Fabrício Farias da Fontoura, Gabriela Roncato, Gisela Martina Bohns Meyer, Cássia da Luz Goulart, Fernanda Brum Spilimbergo, Gerson Cipriano Junior, Marilia Gabriela Bernadeli, Katya Rigatto, Danilo Cortozi Berton

**Affiliations:** ^1^ Programa de Pós‐graduação em Ciências Pneumologicas Universidade Federal do Rio Grande do Sul Porto Alegre Brazil; ^2^ Centro de Hipertensão Pulmonar Santa Casa de Misericórdia de Porto Alegre Porto Alegre Brazil; ^3^ Curso de Fisioterapia Universidade La Salle Canoas Brazil; ^4^ Programa de Pós‐graduação em Ciências da Saúde Universidade Federal de Ciências da Saúde de Porto Alegre Porto Alegre Brazil; ^5^ Research Group on Cardiopulmonary Rehabilitation (GPRC) University of Brasilia (UnB) Brasilia Brazil

**Keywords:** exercise capacity, IMT, PImax, pulmonary hypertension

## Abstract

**Background and Objective:**

To evaluate the effects of a high‐intensity inspiratory muscle training on respiratory muscle strength, exercise capacity, dyspnoea, and health‐related quality of life (HRQL) in patients with pulmonary hypertension (PH).

**Methods:**

Single‐blinded, randomised controlled trial 35 women with clinically stable PH (subgroups 1 and 4), functional class II and III, were randomly assigned to a high‐intensity inspiratory muscle training (IMT) or sham training. Daily 8‐week IMT protocol with 50% (IMT‐50%) of maximal inspiratory muscle pressure (PImax) or sham training with a fixed load of 3 cmH_2_O (IMT‐S). Daily training time consisted of 2 cycles of 30 dynamic inspiratory efforts twice daily, 7 days/week, for 8 weeks using an inspiratory threshold‐loading device.

**Results:**

There were significantly greater improvements in the intervention group (17 patients) compared to sham (14 patients) on PImax [Δpost‐pre = 56.4 cmH_2_O (95% CI 63.5 to 49.3) versus IMT‐S 25.2 cmH_2_O (95% CI 33.1 to 17.4), *p* < 0.001]. The intervention group improved their 6MWT [Δpost‐pre, IMT 50% = 33.5 m (95% CI 15.9 to 51.2) vs. IMT‐S −1.1 m (95% CI −26.7 to 24.4), *p* < 0.001]. Dyspnoea perception at the end of the 6‐min walk test (6MWT) and mMRC significantly improved in the IMT‐50% group: Dyspnoea [Δpost‐pre = −1.1 (95% CI −1.5 to −0.7) vs. IMT‐S −0.2 (95% CI −1.3 to 0.9), *p* < 0.001] and mMRC [Δpost‐pre = −0.6 (95% CI −0.8 to 0.3) vs. IMT‐S 0 (95% CI −0.2 to 0.2), *p* < 0.001].

**Conclusions:**

A high‐intensity protocol of IMT improves respiratory muscle strength, exercise capacity, and dyspnoea in symptomatic patients with PH.

**Trial Registration:** The trial was recorded on the Trials registry RBR‐33gm3k (https://ensaiosclinicos.gov.br/rg/RBR‐33gm3k)

## Abstract 2

In patients with pulmonary hypertension, an 8‐week programme of high‐intensity, daily inspiratory muscle training at 50% of the maximal inspiratory muscle strength resulted in a significant improvement of inspiratory muscle strength, exercise capacity, dyspnoea, and health related quality of life.

**Summary**

- In patients with pulmonary hypertension, an 8‐week programme of high‐intensity, daily inspiratory muscle training at 50% of the maximal inspiratory muscle strength resulted in a significant improvement of inspiratory muscle strength, exercise capacity, dyspnoea, and health related quality of life.

## 1 Introduction

Pulmonary hypertension (PH) is a complex disease with high morbidity and mortality, characterised by shortness of breath and exercise intolerance [1]. The impairment in exercise capacity has been linked to health status [2], contributing to anxiety and depression [3]. Despite recent advances in pharmacological management that have improved the prognosis of PH [4], patients still experience significantly reduced exercise capacity, functional class assessment, and health‐related quality of life (HRQL) [5].

The ideal determinants for exercise prescription in PH patients, including frequency, intensity, duration, and modality, remain unclear. Most clinical trials have used high‐intensity exercise programmes incorporating both aerobic and strength components in a supervised environment [6, 7]. However, implementing such programmes may not be easily achievable due to complex logistics and the inability to supervise patients undergoing this treatment modality.

Beyond pulmonary (ventilation‐perfusion mismatch) and cardiovascular abnormalities (reduced cardiac output), exercise intolerance in PH patients is also associated with other common dysfunctions, including musculoskeletal impairment [8], reduced peripheral vasodilatory capacity, and respiratory muscle weakness [9, 10]. Respiratory muscle training has emerged as a component of comprehensive rehabilitation programmes, demonstrating benefits in both volitional and non‐volitional inspiratory muscle strength [6, 7, 11]. Inspiratory muscle training (IMT) may represent an attractive outpatient and long‐term exercise modality, given its feasibility and cost‐effectiveness. IMT has been shown to improve exercise capacity, despite conflicting results regarding its effects on health‐related quality of life (HRQL). However, few studies have specifically investigated the potential benefits of isolated IMT in PH patients [5].

The purpose of this trial was to investigate the effects of a high‐intensity inspiratory daily 8‐week IMT on respiratory muscle strength, exercise capacity, HRQL, and dyspnoea in patients with PH.

## 2 Methods

### 2.1 Subjects

Of the 98 patients with PH diagnostic confirmed by right heart catheterization (RHC), who receive regular treatment at the Pulmonary Hypertension Center of Santa Casa Hospital (Porto Alegre/Brazil) between December 2014 and December 2017, 35 met the inclusion criteria. Inclusion criteria included: clinically stable patients with target PH medications in the preceding 3 months, World Health Organisation (WHO) functional classes II or III classified in Group 1 (PH) or inoperable Group 4 (chronic thromboembolic pulmonary hypertension, CTEPH) [12] according to the 5th World Symposium on PH held in Nice/France [13] and maximal inspiratory mouth pressure [PImax] < 100% of the predicted normal value [14, 15]. The exclusion criteria constituted long‐term oxygen therapy; musculoskeletal, cognitive, neurological, or psychiatric‐psychological disorders that may interfere in the study protocol; history of moderate or severe chronic lung disease; unstable angina or uncontrolled cardiac arrhythmia; syncope or hospitalisation in the last 6 months; and patients who participated in supervised exercise programs in the past 6 months.

The study was approved by the institutional ethics committee and was performed following the Declaration of Helsinki. Written informed consent was obtained from all participants.

### 2.2 Study Design

This randomised controlled clinical trial was designed according to the consolidated standards of reporting trials statement (CONSORT) [16]. Patients were randomly assigned, using a program to generate random numbers, either to the intervention (IMT‐50%) or the sham group (IMT‐S) for an 8‐week protocol. An intervention‐blinded evaluator performed baseline and final assessments. Incremental cardiopulmonary exercise test (CPET) was performed only at baseline.

### 2.3 Procedures

Baseline clinical assessment before IMT included: clinical and anthropometric characteristics, including age, gender, PH aetiology, functional class, haemodynamic data, pharmacological treatments, previous hospitalisation, respiratory muscle strength, HRQL, 6‐min walk test, spirometry, and CPET [17].

#### 2.3.1 Cardiopulmonary Exercise Testing (CPET)

Incremental CPET until exhaustion was performed according to the standardisation of ATS/American College of Chest Physicians (ATS/ACCP) [18]. The test was performed on a cycle ergometer for lower limbs (Lode BV, Groningen, The Netherlands) with a gas analyser (Vmax, SensorMedics), a digital electrocardiograph (CardioSoft Resting ECG, General Electric Company, Helsinki, Finland) and a portable digital oximeter (General Electric Company, Helsinki, Finland). Cycling familiarisation was performed prior to the test. The protocol consisted of a 3 min rest on the bicycle, followed by a 2 min cycling without load. Subsequently, the workload was increased by 5 W/min or 10 W/min depending on age, gender, body mass, and functional class.

#### 2.3.2 Respiratory Muscle Strength

Maximal voluntary respiratory pressures were registered at the mouth using a digital manometer (MVD‐300, Globalmed, Porto Alegre, RS, Brazil) [19]. A 2‐mm orifice in the system kept the glottis open and prevented any interference from pressure produced by facial muscles. Subjects were comfortably seated in a chair with their feet on the ground, their back unsupported, and their trunk at a 90° angle to their hips. A demonstration of how the manoeuvres should be carried out was given and then performed by the subject after the placement of a nose clip. The subjects were instructed to keep their lips sealed tightly around the mouthpiece so that no air could escape.

PImax values were obtained by inspiration from the residual volume. PEmax was obtained by expiration from total lung capacity, using the same methodology applied in inspiration. During the PImax manoeuvre, the subject kept the mouthpiece in the oral cavity only during inspiration, and in the PEmax manoeuvre, only during expiration [20]. The manoeuvres were sustained at maximal force for approximately 1 s, and the highest value was computed from a minimum of five repetitions for each manoeuvre until the three best measurements. The higher measure could not be the last test because of the potential learning effect. The predicted values were defined according to the equations proposed by Neder et al. [14].

#### 2.3.3 6‐Min Walk Test

Exercise capacity was evaluated by the 6‐min walk test (6MWT) on an enclosed 30‐m track according to the ERS/ATS guidelines [19, 21, 22, 23]. Breathlessness and fatigue perception were determined using the modified Borg Scale [24]. The predicted values were calculated according to a Brazilian reference [25].

#### 2.3.4 Daily Life Dyspnoea

The modified Medical Research Council (mMRC) dyspnoea scale was used to grade dyspnoea during daily activities [26].

#### 2.3.5 Health‐Related Quality of Life

The SF‐36v2 questionnaire (*Medical Outcome Study Short‐Form Health Survey* Version 2) was used to evaluate Quality of life. The SF‐36v2 was translated and validated in Brazil, which had been previously used in PH patients [27, 28]. The SF36v2 predicted values were calculated according to the Brazilian reference [29].

### 2.4 Intervention

#### 2.4.1 Inspiratory Muscle Training (IMT)

Patients in the intervention group received IMT with a load of 50% of PImax (IMT‐50%), which was adjusted weekly. The SHAM group received the same instructions; however, the load was fixed at 3 cmH_2_O (IMT‐S). The device used in the study was the POWERbreathe Plus (POWERbreathe, HaB International Ltd., Southam, UK), which incorporates a flow‐independent one‐way valve to ensure consistent pressure intensity. The training session for both groups consisted of two cycles of 30 dynamic and deep inspiratory efforts, with a 1‐min rest interval between cycles, performed twice daily. The total daily training duration for both groups was approximately 14 min. Participants were instructed to perform the training 7 days per week for 8 weeks, wearing a nose clip during all sessions.

PImax was measured weekly at the research centre by an experienced respiratory therapist to assess respiratory muscle strength and adjust the IMT‐50% group load accordingly. The physiotherapists who provided the IMT sessions to patients in both groups were not blinded to the intervention. The compliance rate with the IMT protocol was assessed during the weekly supervised sessions by a diary including information from the week before; the number of breaths/session and any discomfort or difficulty to complete the session were also assessed [30]. A session was considered completed when ≥ 90% of the prescribed breaths (i.e., 60 per session) were performed.

During the baseline assessment, patients received a training session where they were instructed to perform fast and forceful inspirations and encouraged to achieve maximal inhalation and exhalation with every breath (start inhaling from residual volume and to finish their breath as close to total lung capacity as possible) [15]. Because of the increased tidal volume, a decreased breathing frequency was adopted to avoid hyperventilation and the consequent hypocapnia. Throughout the first training session, oxygen saturation, heart rate, arterial blood pressure, breathlessness, and fatigue perception were monitored to ensure the safety of the training. The patients were instructed to stop immediately in case they experienced any significant discomfort during inspiration.

### 2.5 Statistical Analysis and Sample Size Estimation

Data were presented as absolute and relative frequency (percentage) for categorical variables. Shapiro–Wilk test was used to assess the normality of the data distribution. Quantitative variables are given together with the mean and SD, or else with the median and 25th and 75th percentiles (P25–P75) when they did not meet normality criteria. We performed associations between categorical variables with Fisher's exact test. Multiple comparisons with Bonferroni adjustment were performed in the case of significant ANOVA findings.

The within‐and between‐group effect sizes were calculated using Cohen's d coefficient. Changes in the follow‐up were studied performing analysis of variance using a repeated‐measures mixed design (intrasubject) and a one‐factor (intersubject) design for the analysis of PImax values over time. When the sphericity criteria were not followed, the degrees of freedom were corrected using Greenhouse–Geisser's method. A *p*‐value ≤ 0.05 was considered statistically significant. Statistical analysis was performed using SPSS software, v. 22 (IBM) and Stata software, v. 12 (StataCorp).

The sample size was estimated using an independent t‐test (power of 80% and alpha of 0.05) to detect a difference in PImax between interventions of 73.8 ± 17.2 versus 99.9 ± 21.5 cmH_2_O [31] as 9 individuals in each group. A total of 22 participants were planned to be included, assuming a dropout of 20%.

## 3 Results

**FIGURE 1:**
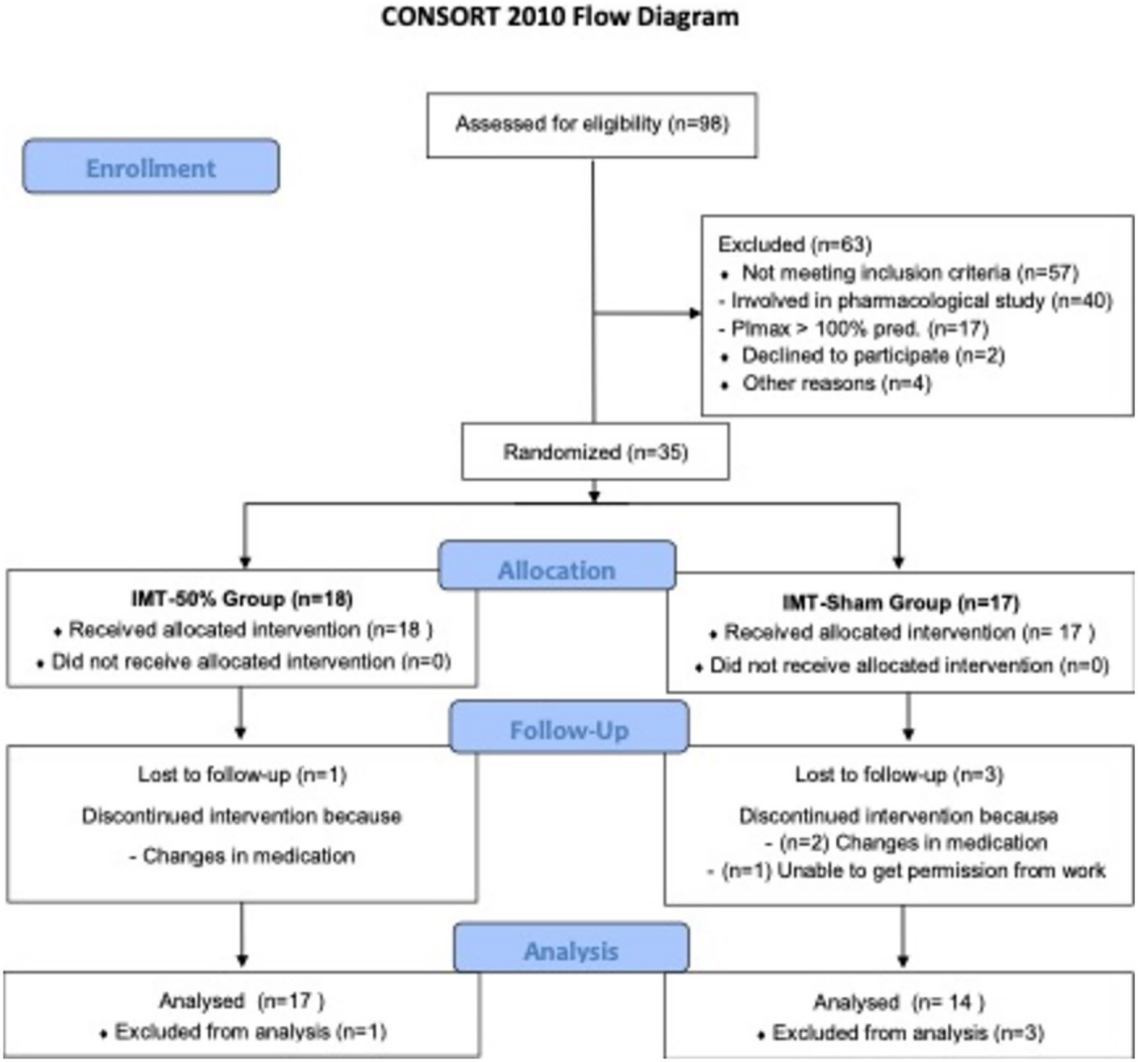
A diagram summarising the flow of participants through the study.

During the 36‐month recruitment period, thirty‐five patients with PH met the study criteria and were enrolled in the trial and divided into two groups. Four patients dropped out during the study; therefore, 31 patients were analysed (Figure 1). Both groups included several PH aetiologies, including idiopathic PH, PH due to congenital heart disease, connective tissue disease, HIV, and thromboembolic PH (only 1 patient in each group).

All subjects were female, and the majority (71%) were in functional class II. There were no significant differences between the groups at baseline (Table 1) and no differences were found in lung function (Table 1) and in CEPT data (Appendix S1 in the Supporting Information). Adherence in both groups were adequate, 77% ± 28% in IMT‐50% and 83 ± 13% in IMT‐S, *p* = ns.

**TABLE 1:** Clinical characteristics.

	All patients (*n* = 31)	IMT‐50% Group (*n* = 17)	IMT‐S Group (*n* = 14)	*p*
Demographics				
Age, y	38.9 ± 8.5	38.8 ± 6.8	41.5 ± 10.6	0.234
BMI, kg/m^2^	25.3 ± 4.2	26.61 ± 4.1	24.67 ± 3.1	0.098
WHO/NYHA				
Class II	22 (71.0)	14 (82.4)	8 (57.1)	0.124^a^
Class III	9 (29.0)	3 (17.6)	6 (42.9)	
PH aetiology				
Idiopathic	11 (35.5)	6 (35.3)	5 (35.7)	0.922^a^
Congenital heart diseases	9 (29.0)	5 (29.4)	4 (28.6)	
Connective tissue disease	6 (19.4)	4 (23.5)	2 (14.3)	
HIV	3 (12.5)	1 (5.9)	2 (14.3)	
CTEPH	2 (12.5)	1 (5.9)	1 (7.1)	
Drug therapy, *n* (%)				
ERA	14 (45.2)	7 (41.2)	7 (50.0)	0.333^a^
ERA + PD5i	9 (29)	6 (35.3)	3 (21.4)	
sGC	6 (19.4)	2 (11.8)	4 (28.6)	
PD5i	2 (6.5)	2 (11.8)	0	
Right heart catheterization				
PAPm (mmHg)	50.3 ± 15.6	48.1 ± 15	53.3 ± 16.4	0.427
SPAP (mmHg)	78.1 ± 23.1	76.2 ± 25.3	82.3 ± 23.1	0.471
Cardiac output (L/min)	5.39 ± 1.7	5.61 ± 1.5	5.14 ± 2.2	0.065
PVR (Wood)	8.43 ± 4.9	7.5 ± 4.2	10.1 ± 5.3	0.218
Cardiac Index (L/min^−1^/m^−2^)	3.18 ± 1.06	3.38 ± 1.18	3.35 ± 1.41	0.440
Pulmonary function test				
FEV_1_ (%)	75.8 ± 15.4	79.1 ± 12.6	73.0 ± 14.6	0.239
FVC (%)	80.4 ± 12.9	82.7 ± 12.2	79.8 ± 14.6	0.147
FEV_1_/FVC (%)	77.8 ± 8.6	79.2 ± 5.4	74.6 ± 6.02	0.364
Respiratory muscle strength				
PI_max_ (cmH_2_O)	−69.4 ± 17.4	−64.2 ± 15.5	−75.3 ± 18.8	0.573
PI_max_ (%)	76.4 ± 18.9	71.8 ± 16.4	82.8 ± 21.0	0.480
PE_max_ (cmH_2_O)	81.1 ± 20.2	80.9 ± 20.2	81.2 ± 20.3	0.850
PE_max_ (%)	88.1 ± 21.9	87.6 ± 22.3	83.8 ± 19.5	0.616
6‐min walking test				
Distance (m)	490 ± 54	499 ± 62	478 ± 43	0.111
Distance (%)	84.5 ± 9.3	87.5 ± 9.8	81.0 ± 7.5	0.033
Dyspnoea final (Borg units)	4.79 ± 2.6 5 [2–7]	4.75 ± 2.8 5 [2–7.5]	4.81 ± 2.4 5 [3.5–7.0]	0.566
Leg effort final (Borg units)	3.93 ± 2.6 3 [2–6]	3.88 ± 2.7 3 [2–6.5]	4.07 ± 2.7 3.5 [2.75–5.5]	0.147
∆ SpO_2_ (%)	−7.68 ± 9.8 3 [14–3]	−5.29 ± 8.5 2 [9–0]	−10.5 ± 10.8 11 [19–0]	0.151

*Note*: All continuous data presented as mean ± SD, *n* (%) or median [IQR]. No significant differences between groups for all variables.

Abbreviations: BMI, body mass index; CTEPH, chronic thromboembolic pulmonary hypertension; ERA, endothelin receptor antagonist; FEV1, forced expiratory volume of 1 s; FEV_1_/FVC ratio, Tiffeneau–Pinelli index; FVC, forced volume vital capacity; HIV, human Immunodeficiency Virus; IMT, inspiratory muscle training; NYHA, New York Heart Association; PAH, pulmonary artery hypertension; PAP, pulmonary artery pressure; PAPm, mean pulmonary arterial pressure; PD5i, phosphodiesterase type 5 inhibitor; PEmax, maximal expiratory mouth pressure; PH, pulmonary hypertension; PImax, maximal inspiratory mouth pressure; PVR, pulmonary vascular resistance; sCG, soluble guanylyl cyclase; SPAP, systolic pulmonary artery pressure; WHO, World Health Organisation.

^a^ Chi‐Square tests.

### 3.1 Respiratory Muscle Strength

**FIGURE 2:**
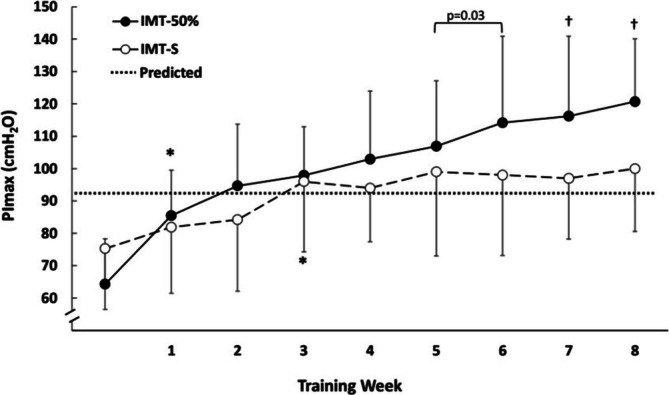
Weekly values of maximal inspiratory pressure (PImax) for the sham (○) and 50% of PImax (•) inspiratory muscle training. The dotted line represents the average predicted value of the PImax (Neder et al. 1999). Data are presented as mean ± SD. *p < 0.001 from baseline in each intervention. †*p* < 0.05 between groups in each week.

Ten patients (59%) in the IMT‐50% group and eight (57%) in IMT‐S had inspiratory muscle weakness at baseline. The improvements in the intervention group (17 patients) were significantly greater compared to sham (14 patients) on PImax (Δpost‐pre = Δpost‐pre = 56.4 cmH_2_O [95% CI (63.5 to 49.3)] vs. 25.2 cmH_2_O [95% CI (33.1 to 17.4), *p* < 0.001]) (Table 2). During the weekly sessions for respiratory muscle strength assessment, patients needed to perform approximately 10 ± 4 repetitions of each manoeuvre (PImax and PEmax) to comply with the repeatability criteria [20]. The number of reacquired repetitions was higher in the first 4 weeks and decreased to 5 ± 2 in the last 2 weeks. Interestingly, 82% of the total PImax increment in the IMT‐S group occurred during the first 3 weeks, 20 cmH_2_O (95% CI: −31 to −9) while the increment of PImax in the IMT‐50% group occurred over the 8 weeks (Figure 2).

**TABLE 2:** Effects of IMT on respiratory muscle strength and functional capacity.

Outcomes	IMT group	Mean ± SD		Within‐group difference in change score pre‐ and post‐intervention	Between‐group difference in change score pre‐ and post‐intervention	
		Pre	Post, 8 weeks	Mean difference (95% CI); Cohen's *d* effect size	Mean difference (95% CI); Cohen's *d* effect size	
Respiratory muscle strength						
PImax (cmH_2_O)	Sham	−75.3 ± 18.8	−100.6 ± 19.4	−25.2 (−33.1 to −17.4); d = −1.324	−31.1 (−41.2 to −21.0); d = −2.319	
	50%	−64.2 ± 15.5	−120.6 ± 19.4	−56.4 (−63.5 to −49.3); d = −3.212		
PImax (%)	Sham	82.8 ± 21.0	108.4 ± 19.7	25.5 (17.1 to 34.1); d = −1.257	38.5 (26.2 to 50.7); d = −2.757	
	50%	71.0 ± 16.3	135.1 ± 25.1	64.1 (54.6 to 73.5); d = −3.028		
PEmax (cmH_2_O)	Sham	81.2 ± 20.3	96.7 ± 19.5	15.5 (7.6 to 23.3); d = −0.778	25.3 (13.8 to 36.7); d = −1.678	
	50%	80.9 ± 20.2	121.7 ± 20.5	40.8 (32.0 to 49.6); d = −2.001		
PEmax (%)	Sham	83.8 ± 19.3	102.8 ± 20.9	18.9 (9.2 to 28.6); d = −0.946	24.6 (11.4 to 37.8); d = −1.638	
	50%	87.6 ± 22.3	131.2 ± 21.7	43.6 (33.9 to 53.3); d = −1.981		
6‐min walking test						
Distance (m)	Sham	478 ± 43	477 ± 62	−1.1 (−26.7 to 24.4); d = 0.018	34.7 (5.8 to 63.6); d = −0.843	
	50%	499 ± 62	533 ± 63	33.5 (15.9 to 51.2); d = −0.543		
Distance predict (%)	Sham	81.0 ± 7.5	81.5 ± 12.2	0.5 (−4.1 to 5.3); d = −0.049	5.0 (0.6 to 10.0); d = −0.722	
	50%	87.5 ± 9.8	93.1 ± 8.4	5.5 (2.7 to 8.4); d = −0.613		
Dyspnoea final (Borg units)	Sham	4.8 ± 2.4	4.6 ± 3.1	−0.2 (−1.3 to 0.9); d = 0.072	0.9 (−0.1 to 1.9); d = 0.490	
	50%	4.7 ± 2.8	3.5 ± 2.7	−1.1 (−1.5 to −0.7); d = 0.436		
Leg effort final (Borg units)	Sham	4.07 ± 2.7	3.07 ± 2.49	−1.0 (−2.6 to 0.6); d = 0.385	0.8 (−2.5 to 1.4); d = −0.321	
	50%	3.8 ± 2.7	3.5 ± 2.9	−0.2 (−1.2 to 0.7); d = 0.107		
mMRC						
	Sham	2.2 ± 0.4	2.2 ± 0.5	0.0 (−0.2 to 0.2); d = 0	−0.6 (−0.9 to −0.2); d = 1.338	
	50%	2.1 ± 0.8	1.5 ± 0.7	−0.6 (−0.8 to 0.3); d = 0.798		

### 3.2 Exercise Capacity and Dyspnoea

The 6MWT distance increased in the IMT‐50% group compared to the sham group: 6MWT [Δpost‐pre = 33.5 m (95% CI 15.9 to 51.2) vs. IMT‐S −1.1 m (95% CI −26.7 to 24.4), *p* < 0.001]. Dyspnoea perception at the end of the 6MWT and mMRC improved significantly in the IMT‐50% group Dyspnoea [Δpost‐pre = −1.1 (95% CI −1.5 to −0.7) vs. IMT‐S −0.2 (95% CI −1.3 to 0.9), *p* < 0.001] and mMRC [Δpost‐pre = −0.6 (95% CI −0.8 to 0.3) vs. IMT‐S 0 (95% CI −0.2 to 0.2), *p* < 0.001] (Table 2).

### 3.3 Health‐Related Quality of Life

Most domains of the SF‐36v2 at baseline assessment were not different between groups. It is important to note that mental health, a significant decrease in all dimensions was observed in both groups. After the intervention, physical functioning (PF), role physical (RF), general health (GH) and vitality significantly improved in the IMT‐50% group (*p* < 0.05) (Table 3).

**TABLE 3:** Comparison of the HRQL changes between the groups.

	IMT group 50% (*n* = 17)				Shaw group (*n* = 14)				
HRQL, Short‐form 36	Brazil Populations Score^c^	Baseline	After (8 weeks)	*p* ^a^	Brazil Populations Score^c^	Baseline	After (8 weeks)	*p* ^a^	*p* ^b^
Physical functioning	82.3 ± 6.3	42.06 ± 22.7	55.5 ± 26.5	**0.001**	83.2 ± 7.2	53.9 ± 20.2	52.5 ± 23.7	0.821	**0.045**
Role physical	82.5 ± 5	42.6 ± 40.2	57.3 ± 37.2	**0.013**	83.9 ± 5.5	63.4 ± 45.2*	71.1 ± 35.1*	0.414	0.438
Bodily pain	78.1 ± 5.4	58.6 ± 24.1	69.8 ± 23.2*	0.083	79.5 ± 6.1	66.8 ± 18.3	76.4 ± 23.2*	**0.039**	0.842
General health	73.7 ± 4.8	40.6 ± 25.7	49.9 ± 28.5	**0.012**	74.8 ± 5.2	44.1 ± 23.1	48.3 ± 27.7	0.285	0.312
Vitality	77.5 ± 7.7	45.3 ± 24	62.9 ± 22.7	**0.0001**	78.4 ± 6.8	55.3 ± 17.9	61.1 ± 20.3	0.143	**0.016**
Social functioning	86.6 ± 3.1	67.6 ± 30.4	72.8 ± 25	0.235	87.2 ± 3.4	78.5 ± 13.3	81.3 ± 18.8*	0.489	0.683
Role emotional	81.6 ± 5.9	62.6 ± 42.2*	69.1 ± 33.2*	0.377	81.4 ± 9.5	73.7 ± 35.1*	81 ± 36.3*	0.545	0.951
Mental health	67.1 ± 11.3	65.4 ± 18.6*	71.2 ± 17.5*	**0.084**	68.9 ± 11	68.5 ± 16.9*	75.7 ± 17.5*	**0.061**	0.771

*Note*: Data are presented as mean ± SD.

Abbreviations: HRQL, health‐related quality of life; SF‐36, Short‐Form 36 Questionnaire.

^a^ Before and after intervention within‐group analysis were used ANOVA and *2‐tailed Student t* test.

^b^ Before and after intervention between group analysis were used *two‐sample t tests*.

^c^ Brazil Populations Score by Laguardia, J. et al. Rev. Bras Epidemiol 2013.

* *p* > 0.05 (in bold).

### 3.4 Adverse Events

Of the 31 enrolled patients in the intervention group, four complained of pain in the scapular waist during the first 3 weeks, and one additional patient presented diffuse muscle pain related to a urinary tract infection. This participant paused the IMT for 7 days in week four. No adverse events were reported in the IMT‐S group. Noticeable no reports of syncope or pre‐syncope, tachycardia, respiratory complications, dizziness, or oxygen saturation < 88% during supervised interventions or home training were noted. No participant dropped out of the intervention group because of IMT‐related adverse events or discomfort.

## 4 Discussion

The present study demonstrated that an 8‐week high‐intensity IMT protocol improved respiratory muscle strength, exercise capacity, dyspnoea as well as functional and general health dimensions of HRQL in patients with Pulmonary Hypertension. To our knowledge, this is the first specific trial to investigate the impact of a daily, high‐intensity and short‐duration protocol of inspiratory muscle training in patients with PH. The main outcome focus was on respiratory muscle strength; however, improvement was also noted in [12], exercise capacity, dyspnoea symptoms, and health‐related quality of life [12].

This study had proposed a daily 8‐week protocol with a duration of approximately 7 min twice daily [31, 32, 33]. The overall load imposed during training is the main factor determining the outcome, suggesting that schemes with similar work intensity may be considered in clinical practice to achieve clinical benefits. Our protocol broadens IMT schemes to be offered based on clinical evidence, reducing by 50% the time spent per day for the training [31, 32]. Furthermore, differently from Laoutaris ID et al. [31] and similarly to Saglam M et al. [32] we compared IMT against a sham intervention, indicating that the differences are not due to a placebo effect.

### 4.1 Respiratory Muscle Strength

The clinical benefit observed in the intervention group (IMT‐50%) was probably related to the structural changes in respiratory muscles. Hypertrophy and an increase in the proportion of muscular type I fibres were described in other populations such as COPD patients training with loads above 50% of PImax in contrast to the sham group without load [34]. Interestingly, patients enrolled in the sham group also experienced some respiratory muscle strength increase during the first 3 weeks, though with lower magnitude. We speculate that the improvement could be related to a resisted muscle training effect of the sham intervention and due to the higher number of PImax manoeuvres performed [34] thus, it is attributed primarily to neural adaptations that occur in the first 5–6 weeks of training [35].

A inspiratory load higher than 50% of the maximal pressure may activate the phrenic nerve, increasing sympathetic vasoconstrictor activity aimed at redirecting blood flow from peripheral to ventilatory muscles [35]. It may explain the lack of improvement in exercise tolerance in patients with heart failure [36] and pulmonary hypertension [31, 32] in previous studies. This hypothesis remains to be tested in future physiological studies including patients with PH.

### 4.2 Exercise Capacity

The patients in the intervention group IMT‐50% increased the 6MWT distance covered by 33.5 m (6.8%) after 8 weeks of the IMT protocol. Current guidelines consider a reduction in the 6MWT of 15% as a negative prognostic marker [12]. On the other hand, improvements in 6MWT distance from baseline have been used as a standard primary endpoint in clinical trials enrolling patients with PH [37]; it reflects an improvement in the quality of life and decreased symptoms [6].

The 6MWD at baseline was 440 m and indicates a low mortality risk in 1 year [12]. This differs from previous [9] studies with PH patients in functional class II–III who covered 376 [32] and 426 m [31] and [12]. Although a “ceiling effect” of 6MWD outcome [23] might have masked the intervention magnitude in our population. Studies with drug therapy considered an improvement of 33–50 m in 6MWT distance clinically meaningful by PH patients [38].

### 4.3 Dyspnoea Symptom

Our results showed a significant increase in exercise capacity with decreased symptoms in the IMT‐50% group. Improved inspiratory muscle strength results in higher pressure generation during rest and exercises tidal volume, which may explain the decrease in perceived respiratory discomfort despite a longer exercise duration, that is, improved neuromechanical coupling [15].

Dyspnoea used to be the main reason to culminate in a worsened perception of the quality of life in this population [3, 38]. Increased exercise capacity with a reduction in dyspnoea perception probably underpins the observed improved HRQL after IMT‐50%. Our findings support previous findings that high‐intensity IMT is a promising strategy to reduce breathlessness [5, 31, 32].

### 4.4 Health‐Related Quality of Life

PH patients present worse HRQL when compared to the general population [12]. Higher daily‐life (mMRC) [39] and exercise dyspnoea (Borg scale) are closely related to worse HRQL, anxiety, and depression [39]. In contrast to Saglam et al. [32], we found a significant improvement in HRQL. They did find a difference only intra‐group in one dominion of the six—emotional reactions. It may reflect the higher reduction in the perception of exertional dyspnoea with attendant improvement in daily life dyspnoea observed in our trial.

### 4.5 Limitations

Similarly, to previous studies [31, 32] this clinical trial included a limited number of patients. Additionally, these factors drive the inclusion of heterogeneous participants to compose a minimal sample size necessary for drawing reliable conclusions. Nonetheless, our results align closely with previous reports, contributing to a growing body of evidence supporting IMT as a promising strategy for reducing morbidity in PH.

The sole recruitment of women limits the generalisability of the findings to the broader population of PH patients. PH manifests differently between genders due to biological, hormonal, and possibly genetic differences. Consequently, the outcomes observed in this study may not be directly applicable to male patients.

This study evaluated only subgroups 1 (Pulmonary Arterial Hypertension) and 4 (Chronic Thromboembolic Pulmonary Hypertension). While these subgroups are clinically relevant, PH is a heterogeneous condition with diverse aetiologies and pathophysiological mechanisms. Therefore, the findings may not be generalisable to other PH subgroups. This is particularly important in the context of increasing survival rates, highlighting the need for further approaches to alleviate the disease burden. Finally, our findings may encourage and provide a foundation for future studies investigating exercise training strategies in larger patient cohorts.

In conclusion, a daily 8‐week IMT‐50% programme resulted in a significant increase in inspiratory muscle strength, leading to improvements in exercise capacity, dyspnoea, and HRQL. However, the long‐term implications and time course of these improvements remain to be determined.

## Author Contributions

**Fabrício Farias da Fontoura:** conceptualization (equal), investigation (equal), methodology (equal), supervision (equal), validation (equal), visualization (equal), writing – original draft (equal), writing – review and editing (equal). **Gabriela Roncato:** conceptualization (equal), data curation (equal), visualization (equal), writing – original draft (equal), writing – review and editing (equal). **Gisela Martina Bohns Meyer:** conceptualization (equal), formal analysis (equal), writing – original draft (equal), writing – review and editing (equal). **Cássia da Luz Goulart:** conceptualization (equal), writing – original draft (equal), writing – review and editing (equal). **Fernanda Brum Spilimbergo:** conceptualization (equal), writing – original draft (equal), writing – review and editing (equal). **Gerson Cipriano Junior:** conceptualization (equal), writing – original draft (equal), writing – review and editing (equal). **Marilia Gabriela Bernadeli:** conceptualization (equal), writing – original draft (equal), writing – review and editing (equal). **Katya Rigatto:** conceptualization (equal), data curation (equal), writing – original draft (equal), writing – review and editing (equal). **Danilo Cortozi Berton:** conceptualization (equal), writing – original draft (equal), writing – review and editing (equal).

## Ethics Statement

The study was approved by the institutional ethics committee and was performed following the Declaration of Helsinki. Written informed consent was obtained from all participants. The trial was recorded on the Trials registry RBR‐33gm3k.

## Conflicts of Interest

The authors declare no conflicts of interest.

## Supporting information


**Appendix S1.** Supporting Information.

## Data Availability

The data used in this study are available upon request from the corresponding author. Interested parties should contact the corresponding author directly to access the data, subject to applicable ethical or legal restrictions.
