# Supplementary material for: Inspiratory Muscle Training on Exercise Capacity, Dyspnoea and Health Status in Pulmonary Hypertension: A Randomised Controlled Trial
Source: Respirology. 2025 May 26;30(8):760–9. doi: 10.1111/resp.70054 (PMC12321694; doi:10.1111/resp.70054)
Supplement: Supplementary file 1 — Appendix S1. Supporting Information. [file RESP-30-760-s001.docx]

| Appendix 1. CPET data. | | | | | | | |
| --- | --- | --- | --- | --- | --- | --- | --- |
| **Cardiopulmonary exercise test** | |  |  |  |  |  |  |
| Peak work rate (W) | 70.5 ± 22.4 |  | 78.7 ± 23.1 |  | 62.6 ± 17.8 |  | 0.106† |
| Peak ⩒O_2_ (L/min) | 0.91 ± 0.31 |  | 1.02 ± 0.28 |  | 0.86 ± 0.32 |  | 0.147† |
| Peak ⩒O_2_ (mL/kg/min) | 14.06 ± 4.91 |  | 15.1 ± 5.53 |  | 12.8 ± 3.39 |  | 0.465† |
| Peak ⩒O_2_ (% predicted) | 57.8 ± 22.05 |  | 58.8 ± 18.9 |  | 56.3 ± 28.8 |  | 0.560† |
| Peak ⩒E/ ⩒CO_2_ slope | 48.5 ± 13.13 |  | 47.7 ± 11.9 |  | 49.8 ± 15.9 |  | 0.328† |
| Peak O_2_ pulse (mL/beat) | 6.36 ± 2.23 |  | 6.39 ± 2.15 |  | 5.95 ± 2.53 |  | 0.501† |
| RER | 1.16 ± 0,16 |  | 1.21 ± 0.18 |  | 1.11 ± 0.11 |  | 0.247† |
| ∆ SpO_2_ (%) | - 4.38 ± 5.98 |  | - 3.25 ± 3.95 |  | - 4.43 ± 7.61 |  | 0.721† |
| Abbreviations: V’O_2_, oxygen uptake; V’E/V’CO_2_, ventilatory equivalent for carbon dioxide; RER, respiratory exchange ratio; ∆SpO_2_, arterial O2 saturation by pulse oximetry (basal-final). All continuous data presented as mean ± SD, n (%) or median [IQR]. No significant differences between groups for all variables. †Independent-samples t-test | | | | | | | |
